# Clinical Lectures on the Gravity and Treatment of Fractures and Wounds by Firearms

**Published:** 1848-09

**Authors:** 


### *Clinical Lectures on the Gravity and Treatment of Fractures and Wounds by Firearms.* By M. Velpeau.

#### ON GANGRENE.

*Gentlemen*,—Gangrene resulting from gunshot wounds is of two kinds: the one produced by a cause *direct;* the other, by a cause *indirect.* The first always exists from the circumstance of the wound itself, and is the result of contusion and of trituration of the soft parts, which are sloughy. It varies in degree: thus it may attack the superficial layers, or the thick parts; it displays itself also more deeply again in the bone than in the soft parts, for these yield under the force of the body producing the wound, whilst the bones which oppose are fractured. This is the gangrene by the cause *direct*, and which necessarily exists.

Gangrene by the cause *indirect* is that of which we shall now more particularly speak. It develops itself under the influence of a wound, and not in the wound itself, which may probably at the time not exist. There are two species of gangrene *indirect;* the first, which develops itself under the influence and from the same cause as the bruise in the organs implicated; the second, which is the result of the inflammation consequent on the wound. The first depends—1, on a lesion of the bone; 2, of the nerves; 3, of the venous and arterial trunks; 4, on injury of the parts.

When a ball traverses a limb and encounters vessels in its passage, many facts may present themselves: the principal artery and its larger branches may be wounded, then gangrene follows almost as a necessary consequence. If the artery alone is wounded, mortification will not supervene.

If the artery and principal vein are wounded at the same time, gangrene will almost inevitably follow, because, supposing that the blood arrives as far as the extremities by the collateral arterial branches, the venous circulation is necessarily interrupted, the blood infiltrates into the tissues, and very soon they become engorged, swollen, and gangrenous.

If there are many large arteries and veins, gangrene will not necessarily follow because an artery and vein have been wounded, for the circulation will be carried on by the others. Thus when the femoral artery and vein have been injured at the same time, gangrene may not result, for the circulation may be established by the profunda artery and vein.

Division of the nerves is a cause of gangrene. In a limb where there are many nerves, as the arm, for instance, the division of one or two nerves is not a cause of gangrene; but, if most of the nerves be injured, paralysis not only follows, but also gangrene.

Division of the skin, whatever may be its extent, never produces this particular disease, no more than lesion of the muscles, provided that the vessels and nerves remain uninjured. Fracture of a bone alone is not a cause of gangrene, but if at the same time the arteries and nerves are divided, and the soft parts are extensively injured, this will inevitably follow.

Gangrene is a complication of gunshot wounds we must notice, but the surgeon will not rest content with mere observation; he will further endeavour to become acquainted with *indications*, to secure as far as possible, the safety of his patients.

Now, the diagnosis of gangrene is easy; it is known that it has certain characteristics by which it cannot be mistaken, but it is by no means easy to foresee and announce its advance. Yet we may, to a certain extent, foretel gangrene from the injuries produced by the projectile. Yet how many difficulties we may have to encounter at the time when we have to decide if such or such an organ has been wounded, or to deduce the probable pathological consequences. For example:—Hemorrhage occurs; it may not often be discovered whether this proceeds from a wounded artery or vein, because the blood, by reason of the contusion of the parts, does not come out in a stream. The division of an artery may not be productive of hemorrhage, because the vessel may be plugged by a hard clot of blood, or much blood may be infiltrated into the surrounding tissues without escaping externally. Where an artery has been wounded two kinds of hemorrhage may occur: either immediate hemorrhage, or hemorrhage produced by the separation of the clot. Thus the surgeon is often very much embarrassed when he attempts to prognosticate the results of the wound as regards hemorrhage.

If the injury includes more or less the substance of the soft parts, and the ball in its course comes in contact with vessels and nerves, it is probable that gangrene will follow.

In fine, when a gunshot wound has been inflicted, it may, to a certain extent, be anticipated whether or not it will be complicated with gangrene; there are cases, however, in which it is impossible to foresee the results. When gangrene has declared itself, whether it has been foreseen or not, two things remain to be accomplished by the surgeon—to determine the prognosis, and then the mode of treatment.

The *prognosis* of gangrene resulting from a gunshot wound is more unfavourable than if the disease resulted from an ordinary wound. Yet spontaneous gangrene is more dangerous than that which follows wounds from firearms, because it depends more frequently upon causes over which the surgeon has no control: thus a spontaneous arterial lesion may be the result of extensive alteration in the organism, &c.; an arterial lesion may, on the one hand, be the result of inflammation of the vessel, which, in consequence, becomes obstructed; on the other, it may be produced by an entire degeneresence of the artery.

Accidental gangrene is not so serious, for though it may depend on a lesion of the vessels, and this lesion is local, yet art may find a remedy.

We have said that gangrene complicating gunshot wounds is more dangerous than that which follows ordinary wounds—and why? Because the projectiles make the soft parts slough, destroy the vessels and nerves, and fracture the bones. The result speedily of this trituration is a *putrilage*, which exercises a baneful influence on the economy, and sets up the commencement of a species of poisoning.

What should be the treatment of gangrene? It must be altogether surgical, and is summed up entirely in the amputation of the diseased parts. Formerly, when ligature of the arteries was not attempted, it was thought better to leave the dead parts to detach themselves and drop off; but, now it is known how to tie the vessels, amputation is preferred, because, in leaving the gangrenous parts to drop off spontaneously, a bad stump is the consequence, and the bone protrudes in the middle of the flesh; for at the bone the mortification does *not* go so far as in the soft parts. This, as may be expected, produces great inconvenience. It is needful, therefore, when gangrene supervenes, to have recourse to amputation: this is imperative on us if the general health is good; a commencement of infection, however, will be a formal contraindication.

Here, gentlemen, the important question presents itself: is it proper to amputate immediately when the gangrene has manifested itself; or is it proper to wait till its progress is arrested? The surgeons who advocate the latter opinion do it for the following reasons:—Gangrene, they say, produces gangrene; it is never known where it will stop, consequently it will attack the stump.

The vessels are obliterated, not only at the surface of the wound, but even within, to a distance which cannot be determined; and this obliteration may extend through the large vessels into the splanchnic cavities. Moreover, in gunshot wounds there is a shock, a stupor, and an impoisonment—three things which make it most uncertain when the gangrene will be arrested—from which it follows that it is by no means proper to amputate before there are indications of its progress being staid. These are the principal arguments of those who are opposed to an operation before the disease is arrested.

Gentlemen, it is proper here to consider the question—What is the cause of gangrene? When it depends on spontaneous lesion of the arteries it is certainly far better to wait till there are indications of its ceasing to advance, because we are not certain when the arterial lesion will be arrested.

But if the gangrene is traumatic—if the vessel has been opened, and there is no reason to suppose that it is diseased above the wound—it is not necessary to wait for the separation of the parts, for the modification will still go on. The presence of a gangrenous part excites, in the living tissues adjacent, an inflammatory and eliminatory reaction; and the organism, infected by a double source of poison cannot sustain the struggle of the living tissues with the dead, and the system will very soon be exhausted. Thus, in a general way, and *à priori*, it will be better, in gunshot wounds, to amputate at once. Yet this is not an invariable rule.

If, for example, as M. Larry has asserted, the gangrene commences at the extremities of a limb, near the fingers, we must wait till its progress is arrested; but if it show itself in the thigh, or in the substance of the arm, immediate amputation, before the separation of the dead parts, is the only chance of safety which remains to the patient.

Those also who have been operated on before gangrene has been arrested, are found in an unfavourable condition. In general, as you may observe in two men who have lately undergone amputation, the one of an arm the other of the leg, in both of which the gangrene was not arrested, that the stumps are flabby, suppuration is established with difficulty, and the whole system appears to suffer. These unfavourable conditions exist more rarely when the disease is left to take its own course. The conclusion, then, at which we arrive is, that it is necessary very frequently to amputate before the dead parts separate, lest in temporizing the favourable opportunity may pass away, and thus the only chance of saving the patient be lost; for often, the longer an operation is deferred the more impracticable it becomes.—*London Med. Times.*

